# Languages and future-oriented economic behavior—Experimental evidence for causal effects

**DOI:** 10.1073/pnas.2208871120

**Published:** 2023-02-06

**Authors:** Ian Ayres, Tamar Kricheli Katz, Tali Regev

**Affiliations:** ^a^Yale Law School, New Haven, CT 06511; ^b^Tel Aviv University, Buchmann Faculty of Law, 6997801 Tel Aviv, Israel; ^c^Tiomkin School of Economics, Reichman University, 4610101 Herzliya, Israel

**Keywords:** languages, economic behavior, future time reference, discount rate

## Abstract

Studies have shown that the use of languages which grammatically associate the future and the present tends to correlate with more future-oriented behavior. We take an experimental approach to go beyond correlation. We asked bilingual research participants, people fluent in two languages (12 language pairs) which differ in the way they encode time, to make a set of future-oriented economic decisions. We find that participants addressed in a language in which the present and the future are marked more distinctly tended to value future events less than participants addressed in a language in which the present and the future are similarly marked. In an additional experiment, bilingual research participants (seven language pairs) were asked to choose whether they wish to complete a more enjoyable task first or later (delayed gratification). When addressed in a language in which the present and the future are marked more distinctly, participants tended to prefer immediate gratification more than when addressed in a language in which the present and the future are marked less distinctly. We shed light on the mechanism in a within-person experiment in which bilingual research participants (nine language pairs) were asked to spatially mark the distance between the present and the future. When participants were addressed in a language in which the present and the future are marked more distinctly, they tended to express more precise temporal beliefs compared with when addressed in a language in which the present and the future are marked less distinctly.

## Significance

Our results suggest that the time-related schemas embedded in languages are easily and immediately activated; asking the same payment questions in a different language resulted in different time preferences for otherwise similar participants as well as in differences in the tendency to delay gratification. Asking people to mark the distance between the present and the future in a different language resulted in differences in the precision of the perceived distance.

Do languages affect the attitudes, preferences, and behaviors of the people who speak them?

Numerous studies have documented correlations between the linguistic features and grammatical structures of languages and the attitudes, preferences, and behaviors of the people who speak them. Thus, for example, it has been shown that speakers of languages with different structures and features differ accordingly in their processing of colors, future-oriented economic behavior, and gendered attitudes (1–8). However, evidence for the causal effects of the features and structure of languages on attitudes, preferences, and behaviors is harder to establish. It is a challenge to demonstrate empirically that using a specific language can affect and not just merely reflect or correlate with the way we perceive the world. Indeed, scholars across several disciplines have debated, and continue to debate, the relationship between language and thought (8). Some have argued that languages do not restrict people’s perceptions and behavior (9), whereas others (who subscribe to the linguistic relativity hypothesis) have asserted that speakers of languages develop language-specific schemas and structures which affect their perceptions and behavior (10–18).

We contribute to this long-standing debate by providing evidence for the causal impact of the encoding of time in the language spoken on the intertemporal economic choices that people make and on the precision of their temporal beliefs. Our findings suggest that perceptions of time are differently embedded in languages and can impact everyday human behavior.

Languages vary in the ways in which they encode time. In some languages, like German, the same grammatical tense is often used to refer to both present and future events (“futureless languages”; “weak-FTR languages”). Other languages, like English and French, have the obligatory grammatical marking of the future tense (“futured languages”; “strong-FTR languages”).^*^

Studies based on survey data show that the use of futureless [weak future time reference (weak-FTR)] languages, which grammatically associate the future and the present, tends to correlate with more future-oriented behavior on the part of people and organizations. Thus, for example, across and within countries, speakers of such languages save more, retire with more accumulated wealth, smoke less, practice safer sex, are less obese, and care more about the environment (1, 19–26).

Why would speakers of weak-FTR languages express more future-oriented economic behavior?

One possible explanation is that consistently speaking about future events in the present tense can make the future seem more immediate and less distant (the “distance hypothesis”). Thus, the speakers of weak-FTR languages may tend to view the future as less distant and as a result value future rewards more than the speakers of languages in which the present and the future are distinctly marked (26)(1).

**Fig. 1.**
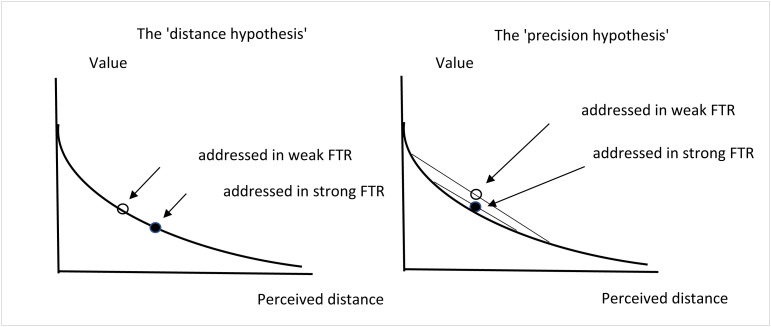
Time discounting and the “distance” and “precision” hypotheses.

Another possible explanation is that because weak-FTR languages do not obligate speakers to mark present and future events differently, speakers of these languages might not think as precisely about the temporal distance of future events as speakers of strong future time reference (strong-FTR) languages (the “precision hypothesis”). In other words, speakers of weak-FTR languages might less finely divide the temporal space between the present and the future than speakers of strong-FTR languages. As a result, speakers of weak-FTR languages may tend to more fuzzily distinguish between the present and the future. Relatedly, speakers of weak-FTR languages might vary more in their temporal beliefs compared with speakers of strong-FTR languages. Because people’s time discounting function tends to be convex, speakers of weak-FTR languages may therefore tend to discount future rewards less than speakers of strong-FTR languages, individually or on average (26). Fig. 1 illustrates the two hypotheses (for a similar illustration, see ref. 26, figure 2.1).

Note that perceptions of distance and the precision of temporal beliefs might be directly activated by the linguistic future time reference (FTR) or indirectly activated by the evocation of the cultural beliefs about time associated with the language and its FTR. In other words, being addressed in a strong-FTR language might directly affect people’s perception of how distant the future is or how precise their temporal beliefs are. Alternatively, being addressed in a strong-FTR language might indirectly activate cultural beliefs about time associated with the language that might result in viewing the future as more distant or expressing more precise temporal beliefs.

Finally, a third possible explanation for the correlation found between languages and future-oriented economic behavior is that cultural differences regarding time preferences across and within countries (27) might be reflected both in the languages spoken and in the observed differences in the speakers’ future economic behavior (the “correlation hypothesis”).

Although survey data do provide the opportunity to identify correlations between the way in which a language encodes time and the future-oriented economic behavior of the people who speak it, it is hard to use it as a means of determining a “causal effect” between the language spoken and future-oriented economic behavior. In other words, it is nearly impossible to hold constant the unmeasured cultural differences, across and within countries, that might be reflected both in the language spoken and in the observed differences in the future economic behavior of its speakers. Indeed, following Chen’s (1) research, some studies have argued that the research merely shows that the languages we speak reflect the societies and cultures in which we live but does not show that the languages we speak influence our perceptions and behaviors (28–31).

Weighing in on the debate, Sutter et al. (32) studied differences in the intertemporal choices of children living in a bilingual city where about half of the inhabitants spoke German (a weak-FTR language) and the other half spoke Italian (a strong-FTR language). Using an intertemporal choice experiment, they found that German-speaking primary school children were more likely than their Italian-speaking peers to delay gratification. However, like Chen’s study, their study could not rule out the possibility that the observed differences in behavior were generated by the cultural differences between the two groups. Galor et al. (33) sought to provide evidence for the causal effect of the encoding of time in a language on its speakers’ educational attainment. To do so, they exploited variations in the native languages spoken by the children of migrants with identical ancestral countries of origin; they were able to show the significant positive effect of speaking a futureless language on educational attainment. Because the analysis compared children with the same ancestral countries of origin, the experiment’s design controlled for all the cultural differences associated with countries of origins. Yet, it is not possible to fully control for the cultural differences associated with subcultures within countries (those reflected in languages within countries).

Finally, in one recent study, a different approach was taken to address the relationship between the obligation to use the future tense and future-oriented economic behavior. Instead of focusing on the distinction between strong- and weak-FTR languages, the researchers have focused on the effects of participants’ tendency to use the future tense within a language. It was found that a higher use by participants of the future tense within a language (English or Dutch) resulted in less—not more—temporal discounting (34). These interesting—within language—findings point to an additional mediating mechanism; the grammatical obligation to use the future tense in strong-FTR languages tends to oblige speakers to use terms like “will” that denote the high certainty of future events (modal notions of high certainty). As a result, speakers of strong-FTR languages may perceive the certainty of future events as greater than speakers of weak-FTR languages.

We take a different methodological approach to identifying the causal effect of language on future-oriented behavior and to shed light on the mechanisms. We wish to show that the encoding of time in a language not only reflects but also generates differences in future-oriented economic behavior. Thus, we hypothesize that when people are addressed in a futured language, one that grammatically differentiates between the present and the future—i.e., it has a strong-FTR—they tend to discount future events more. This, in turn, encourages less future-oriented behavior, such as spending more in the present and preferring more immediate gratification. When people are addressed in a futureless language, one that does not differentiate grammatically between the present and the future—i.e., it has a weak-FTR—they discount the future less. This, in turn, encourages more future-oriented behavior, such as saving for the future and delaying gratification.

We also wish to show that perceptions of time are differently embedded in languages and are activated when the language is spoken. In doing so, we test both the distance hypothesis and the precision hypothesis. First, we test whether when people are addressed in a futured language (a strong-FTR language), they tend to view the future as more distant compared with when addressed in a futureless language (a weak-FTR language) (the distance hypothesis). Then, we test whether when people are addressed in a futured language (a strong-FTR language), they express more precise temporal beliefs than when addressed in a futureless language (a weak-FTR language) (the precision hypothesis).

To do so, we conducted three experiments. The Yale University Institutional Review Board approved all three studies. In all studies, informed consent was obtained online after participants were directed to Qualtrics. All participants consented. (All data and codes are available at (35)). The first study focused on discounting of future rewards. In a between-subject study, we asked bilingual people, fluent in two languages which differ in the way that they encode time, to make a future-oriented economic decision: Specifically, we asked participants, in one of the two languages in which they are fluent, to make a set of binary choices about whether they wished to be paid a certain amount of money earlier (today) or a larger amount of money later (the following week). We then tested whether the people randomly assigned the question in a strong-FTR language required more by way of future compensation than those asked the question in a weak-FTR language.

Following the EUROTYP project (36) and Chen (1), we separated the languages we explored into two broad categories: weak- and strong-FTR. Strong-FTR languages are those that require future events to be grammatically marked when making predictions. Weak-FTR languages do not require such grammatical marking.

Altogether, in the first study, we used 12 language pairs, in which one language uses the same grammatical tense for the present and the future—i.e., it has a weak-FTR—and the other has a strong-FTR.

The second study focused on delayed gratification. We asked bilingual people, fluent in two languages which differ in the way in which they encode time, to fulfill two tasks; one task was presented as more enjoyable and the other as more tiring. Participants were addressed in one of the two languages in which they are fluent and asked to choose with which of the two tasks they would like to start. After fulfilling the two tasks, participants were addressed in the other language in which they are fluent and were asked to make a similar choice between two additional tasks. Altogether, in the second study, we used seven language pairs, in which one language has a weak-FTR—and the other has a strong-FTR.

The third study focused on the mechanisms. In a randomized within-subject study, we asked bilingual people, fluent in two languages which differ in the way in which they encode time, to mark the distance between the present and the future: Specifically, we asked participants, in one of the two languages in which they are fluent, to spatially mark the distance between “now” and “later” and then in the other language to spatially mark the distance between “today” and “tomorrow.” Altogether, in the third study, we used eight language pairs, in which one language has a weak-FTR—and the other has a strong-FTR.

## Study 1—Discounting Future Rewards

The experiment involved bilingual participants proficient in one weak-FTR language (German, Dutch, and Mandarin) and one strong-FTR language (English, French, Spanish, and Hindi). (Hypotheses were described before running the study in an Institutional Review Board (IRB) application that is available at (35).

The experiment was conducted in the spring and summer of 2019. Participants, recruited via Amazon Mechanical Turk (MTurk) [a crowdsourcing marketplace for Human Intelligence Tasks (HITs)], were randomly assigned to either the weak- or strong-FTR experimental condition. They were first asked (in either the randomly assigned weak- or strong-FTR language) to make a set of binary choices about whether they wished to be paid a certain amount of money earlier ($3 today) or a larger amount of money later (the following week). We asked the participants to make a choice with eleven binary decision problems varying the value of the future compensation to be received in a week from $3.05 to $7. This procedure builds upon previous studies, in which similar multiple price list procedures were used to elicit the participants’ discount rates, i.e., the amount for which participants were willing to receive a delayed payment (37, 38). After choosing their preferred payment methods, participants were asked about their level of comfort in both languages and their country of residence. They were then asked to take language proficiency tests in the two languages in which they had declared themselves to be proficient. Each language proficiency test consisted of nine questions. The order of the two proficiency tests was randomized. (The words used in each language, payment options, and proficiency tests are provided in *SI Appendix*.) After completing the two proficiency tests, participants were asked a series of demographic questions. The geolocation of the participants was also coded. Participants were then given instructions on how to receive payment. The results of participants who proved not to be proficient in both tested languages were excluded from the study.

The experiment therefore consisted of 12 subexperiments (4 strong-FTR languages × 3 weak-FTR languages) × 2 experimental conditions (compensation question in strong-FTR or weak-FTR language). Over the course of 8 mo starting in November 2018, we published 12 different HITs on MTurk, inviting bilingual participants to participate in our study if they were genuinely fluent in the two languages.

Altogether 6,189 participants declared themselves to be bilingual and fluent in the two languages, but only 3,804 completed the experiment. Only 717 of the participants passed the two language proficiency tests assigned to them (i.e., received a score of at least six of nine in each of the proficiency tests). We also excluded participants who displayed inconsistent time preferences and those who participated from the same Internet Protocol (IP) address as other participants. The final sample used in the analysis consisted of 565 participants (Table 1).

**Table 1.** Attrition

	Weak-FTR		Strong-FTR		Difference
	Observation	Proportion	Observation	Proportion	*P*-value
Step					
Assignment to treatment	3,033		3,156		0.118
Answered payment questions	1,980	0.65	2,213	0.70	0.000
Completed survey	1,822	0.60	1,982	0.63	0.027
Proficient in both languages	364	0.12	353	0.11	0.316
Unique users	310	0.10	295	0.09	0.247
Consistent time preference	289	0.10	276	0.09	0.285

Of the 565 participants, 289 were assigned to the weak-FTR condition and 276 to the strong-FTR condition. Table 2 presents the sample characteristics by the experimental condition.

**Table 2.** Sample characteristics by experimental condition

	Weak-FTR	Strong-FTR
Language pairs		
English–Dutch	0.13	0.11
English–German	0.21	0.23
English–Mandarin	0.15	0.17
French–Dutch	0.04	0.05
French–German	0.06	0.10
French–Mandarin	0.04	0.03
Hindi–Dutch	0.05	0.05
Hindi–German	0.08	0.04
Hindi–Mandarin	0.05	0.03
Spanish–Dutch	0.06	0.05
Spanish–German	0.10	0.08
Spanish–Mandarin	0.04	0.05
Payment reservation price		
$3.05	0.33	0.30
$3.25	0.11	0.11
$3.50	0.07	0.08
$3.75	0.06	0.04
$4.00	0.13	0.13
$4.50	0.06	0.08
$5.00	0.10	0.08
$5.50	0.02	0.02
$6.00	0.02	0.04
$7.00	0.03	0.05
None selected	0.06	0.09
Proficiency in the addressing language	7.45 (1.13)	8.15 (1.09)
Strong–weak proficiency gap	0.51 (1.15)	0.54 (1.20)
Female	0.38	0.37
White/Caucasian	0.41	0.41
African American	0.02	0.02
Hispanic	0.05	0.06
Asian	0.48	0.47
Other Race	0.04	0.04
College	0.78	0.79
Strong language genus		
Germanic	0.48	0.51
Indic	0.18	0.12
Romance	0.33	0.37
Weak language genus		
Germanic	0.28	0.28
Indic	0.72	0.72
N	289	276

The table reports group means. SEs are in parentheses.

## Results

Table 3 presents the results of Tobit regression models predicting participants’ lowest accepted delayed payment value. For each participant, we capture the lowest amount for which they indicated a preference to be paid a week from now rather than being paid $3 immediately. Participants who provided inconsistent time preferences were excluded from the analysis.

**Table 3.** Tobit regression models predicting lowest accepted delayed payment

	(1)	(2)	(3)	(4)	(5)
Asked in strong-FTR	0.504*** (0.145)	0.497*** (0.145)	0.276^†^ (0.149)	0.521*** (0.143)	0.515*** (0.144)
Proficiency in the addressing language	0.437*** (0.063)	0.431*** (0.063)	0.603*** (0.073)	0.451*** (0.066)	0.433*** (0.066)
Strong–weak proficiency gap		0.118* (0.056)	−0.189* (0.085)	0.077 (0.061)	0.074 (0.059)
Asked in strong-FTR × strong–weak proficiency gap			0.607*** (0.130)		
Female				0.158 (0.141)	0.177 (0.142)
African American				0.519 (0.447)	0.591 (0.453)
Hispanic				−0.234 (0.316)	−0.233 (0.312)
Asian				0.580*** (0.178)	0.644*** (0.174)
Other				−0.036 (0.383)	−0.071 (0.387)
College graduate				−0.239 (0.174)	−0.266 (0.175)
Language pair dummies				Y	
Strong-FTR Genus Indic					−0.133 (0.219)
Strong-FTR Genus Romance					−0.105 (0.161)
Weak-FTR Genus Indic					−0.186 (0.168)
Constant	6.744*** (0.472)	6.638*** (0.473)	8.086*** (0.564)	7.165*** (0.565)	7.272*** (0.570)
Sigma	2.162*** (0.186)	2.148*** (0.184)	2.062*** (0.177)	1.949*** (0.168)	2.021*** (0.175)
N	523	523	523	509	509
Pseudo R^2^	0.032	0.035	0.049	0.063	0.054

^†^<0.1, *p<0.05, **p<0.01, ***p<0.001

We use Tobit (censored) regression models because the dependent variable is left and right censored; our sample includes participants for whom it was impossible to determine their precise preferences [those who denied all delayed payment offers (31.2% of participants) and those who accepted all delayed payment offers (7.4%)].

In model 1, we estimate the effect of the experimental condition controlling for the participants’ proficiency in the language in which they were asked the payment questions. In model 2, we add the gap in the participants’ proficiency in the strong, compared with the weak, FTR language. The gap reflects the participants’ relative immersion in the strong, compared with the weak, FTR language and culture and captures the correlations observed by Chen (1). Model 3 includes an additional interaction between the gap in proficiency and the experimental conditions. Model 4 includes the demographic characteristics of the participants and the language pair in which the participants were bilingual. Finally, following Roberts et al. (31), Model 5 controls for the origins of the languages in which the participants are fluent (and thus does not include the specific language pair).

As predicted, being addressed in the strong-FTR language generated a higher time discount rate than being addressed in the weak-FTR language. In models 1, 2, 4, and 5, the lowest accepted delayed payment for participants addressed in a strong-FTR language is at least 50 cents higher than the lowest accepted delayed payment for participants who were addressed in the weak-FTR (*P* < 0.001). The effects of being addressed in the strong-FTR language, in all the models, are statistically significant. Participants’ proficiency in the language in which the payment questions were asked also affected their preferences. More proficient participants had lower time discount rates compared with the less proficient participants. This may be because the more proficient participants understood the payment questions better, or because more fluent participants are also more willing to delay immediate reward, compared with less proficient participants (scores in the two proficiency tests were positively correlated).

The strong–weak gap variable in models 2 and 3 captures participants’ relative proficiency in the strong, compared with the weak, FTR language. It thus captures the relative immersion of participants in the strong-FTR language and culture. The significant and positive effect in model 2 (*P* < 0.05) implies that participants who are relatively more proficient in the strong-FTR language (compared with their proficiency in the weak-FTR language) tended to have a higher time discount rate compared with participants who are relatively more fluent in the weak-FTR language.

The significant and positive interaction in model 3 (asked in the strong-FTR * strong–weak gap, *P* < 0.001) suggests that the effects of being asked the payment questions in the strong-FTR language are significantly stronger for participants who are more proficient in the strong-FTR language.

Finally, for robustness, we replicate the analyses while controlling for the participants’ self-reported native language (in addition to the proficiency gap we observed). We also replicate the analyses with country of residence fixed effects (so as to control for the differences in the impact of $1 on the participants’ well-being). Results remain similar in magnitude and statistical significance.

In *SI Appendix* (*SI Appendix*, Tables S1 and S2), we present the results of ordinary least squares (OLS) and ordered logit regression models predicting participants’ reservation prices. The results of these models are similar to the results presented in Table 3.

Note that our experimental design enabled us to hold constant some of the mediating effects of certainty “modal” terms like “will” (high-certainty modal term) or “may” (low-certainty modal term). When presenting participants with the option to be paid a larger amount of money later (the following week) in strong-FTR languages, we used high-certainty “modal” terms. Thus, the higher discount rates of participants who were addressed in strong-FTR languages cannot be attributed to the usage of specific low-certainty “modal” terms. Moreover, the findings suggest that even when high-certainty “modal” terms are used, participants tend to discount future events more when addressed in strong- compared with weak-FTR languages.

### Attrition and Selection Bias.

Only 61% of the participants who started the experiment completed it (Table 1). Our concern is that participants who were asked the payment questions in a language in which they were less proficient tended to quit the study more, which is to say, participants who were more fluent in the strong-FTR language and were asked the payment questions in the weak-FTR language were more likely to leave the experiment than those who were more fluent in the strong-FTR language and were asked the payment questions in the strong-FTR language.

If this indeed was the case, the attrition would generate a biased sample, in which the participants who were asked the questions in the strong-FTR language would also be more proficient in the strong-FTR language compared with the participants who were asked the questions in the weak-FTR language. If the sample were indeed biased, it would be impossible to disentangle the effects of the experimental condition (being asked in the strong-FTR language) from the effect of one’s proficiency in the strong-FTR language.

In *SI Appendix*, Table S3, we present the results of balancing tests comparing the characteristics of participants by experimental condition.

We can see that participants who were asked the payment questions in the strong-FTR language are not significantly more fluent in the strong-FTR language compared with the participants who were addressed in the weak-FTR language. Yet, the findings suggest that participants who were fluent in French (a strong-FTR language) were disproportionately represented in the strong-FTR experimental condition. To eliminate the concern that this imbalance generated the results that we observed, we estimate the same Tobit regression models predicting participants’ lowest accepted delayed payment after excluding all the French-speaking participants from the sample. The results we obtain are very similar to the results that are obtained with the full sample (*SI Appendix*, Table S4). This suggests that it was not the attrition from the experiment that generated the results we report. Finally, the results of the balancing tests presented in *SI Appendix*, Table S3 further suggest that people who were fluent in Hindi (a strong-FTR language) were disproportionately “underrepresented” in the strong-FTR condition. Although this bias should decrease the probability of obtaining the results we report, we also estimate the same Tobit regression models predicting participants’ lowest accepted delayed payment after excluding all the Hindi-speaking participants from the sample. The results we obtain are not statistically different to the results presented in Table 2 (*SI Appendix*, Table S2).

### The English Language.

Following Chen (1), we coded the English language as having a strong-FTR. However, critics have argued that English does not have an obligatory grammatical marking of future events. To eliminate the concern that participants who were addressed in English generated the results we observed, we estimate the same Tobit regression models predicting participants’ lowest accepted delayed payment after excluding all the English-speaking participants from the sample. The results we obtain are very similar to the results obtained with the full sample (*SI Appendix*, Table S5).

Following this concern, and to rule out the possibility that any other language or pair of languages may have driven the results we observed, we repeat the analyses on subsamples of the data, excluding one language or pair of languages at a time. The effects remain similar in magnitude and statistical significance.^†^ We therefore conclude that the effects we observed are not generated by one of the languages or by one pair of the languages that we studied.

Our first study has some limitations. Most notably, it is possible that the languages we chose are associated with cultural scripts, such as cultural scripts regarding trust, the strength of the economy, or norms about savings. One such possibility is that being addressed in a futureless language makes the future more immediate and therefore increases people’s trust in others (39). In our first study, this would imply that the research participants who were addressed in the futured language thought more about the possibility that payment might not be received. Another possibility is that the strong-FTR languages we chose (English, French, Spanish, and Hindi) are associated with countries with less wealthy economies than the countries associated with the weak-FTR languages we chose (Mandarin, Dutch, and German). To eliminate this concern, we reran our analysis on a sample which excluded Hindi and Spanish speakers. Results remained similar in magnitude and statistical power. In addition, in order to reduce these and related concerns and to provide direct evidence for the mechanism that generated the results we observed, we supplemented the analysis with two additional experiments.

## Study 2—Delayed Gratification

The second experiment was designed to explore whether people tended to delay gratification more when addressed in a strong-FTR language compared with a weak-FTR language. It involved bilingual participants proficient in one weak-FTR language (German, Dutch, and Mandarin) and one strong-FTR language (English, French, and Spanish^‡^). The experiment was conducted in the spring of 2022. Participants were targeted via Facebook ads and directed to our Qualtrics survey. The experiment involved two stages, one in each language (the weak- and strong-FTR language). Each stage involved first taking a proficiency test and then being introduced with two tasks; one task was presented as more enjoyable and the other as more tiring. Participants were then asked to choose with which of the two tasks they would like to start. After fulfilling the two tasks, participants were addressed in the other language in which they are fluent and were asked to fulfill a proficiency test and to make a similar choice between two additional tasks.

One set of tasks (set A) included the following two tasks: 1) watching a funny short clip and answering a question about it and 2) watching a short clip and counting the number of cars presented in it. The other set of tasks (set B) included the following two tasks: 1) looking at award-winning photographs of nature and picking those that the participants liked the most and 2) carefully counting the number of times the digit “1” appears in a table of 480 numbers. Before making their choices, participants were informed that each task should take about 1 min. The order of sets presented to the participants was randomized. The words used in each language and proficiency tests are provided in *SI Appendix*.

The final sample for the study included 598 participants who received scores higher than 6 of 9 in the two proficiency tests they took (the answers of participants with lower proficiency scores were not recorded, and they were not able to complete the study). In *SI Appendix*, Table S6, we report the sample characteristics. In *SI Appendix*, Table S7, we report the number of participants in each language pair we used in the experiment.

Although the design of the experiment was a within-person research design, the statistically significant results we observe and report apply only to the first choices participants made (between-person results). In other words, the within-person results we obtain are statistically nonsignificant, perhaps because of the experimental design that required participants to first fulfill the two tasks (in their preferred order) and only later to make their second choices.^§^

**Fig. 2.**
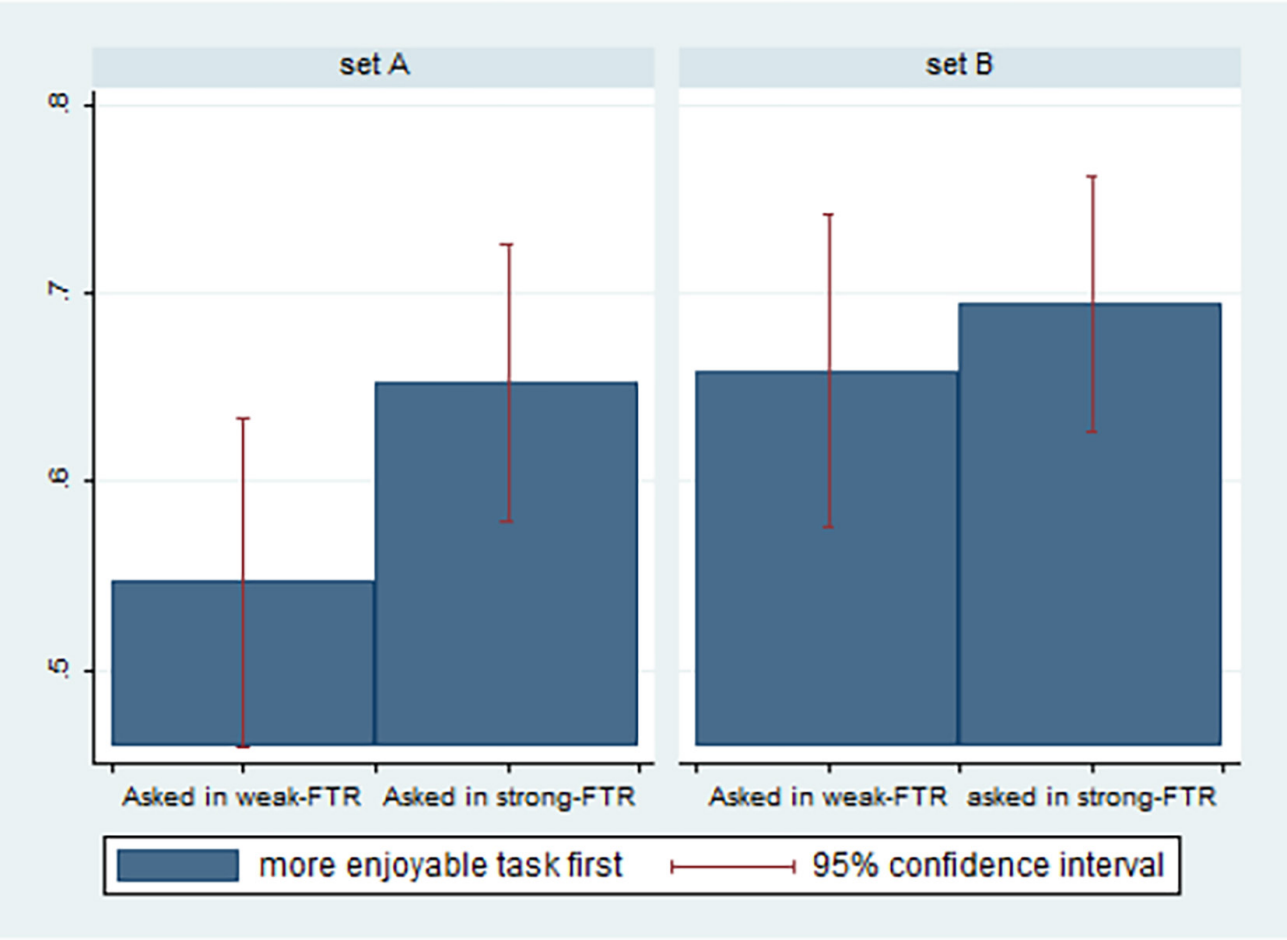
Immediate gratification by experimental condition and nature of tasks (first choices, study 2).

In the first stage of the experiment, 64% of the participants preferred to start with the task that was presented as enjoyable; when addressed in the strong-FTR language, 67% of participants preferred to start with the more enjoyable task, whereas when addressed in the weak-FTR language, only 60% of the participants preferred to start with the more enjoyable task (*P* < 0.05).^¶^ Fig. 2 presents the differences in the expressed preferences by experimental condition and by the nature of the tasks presented (set).

In *SI Appendix*, Table S8, we report the results of OLS regression models predicting participants’ preference for starting with the more enjoyable task by whether questions were asked in the strong- or weak-FTR language and additional controls. The sample includes only participants’ first choices. In all models, being asked to choose the first task in a strong-FTR language increases the tendency to prefer starting with the more enjoyable task by at least 0.068 (*P* < 0.01). Similar effects are obtained when logistic regression models are estimated.

## Study 3—Mechanisms

The third experiment was designed to explore the mechanisms driving the results we obtained in studies 1 and 2. It tested whether people viewed the future as being more distant when addressed in a strong-FTR language compared with a weak-FTR language (the distance hypothesis) and whether people express more precise temporal beliefs when addressed in a strong-FTR language compared with a weak-FTR language (“the precision hypothesis”).

The experiment involved bilingual participants proficient in one weak-FTR language (German, Dutch, and Mandarin) and one strong-FTR language (English, French, and Spanish^#^). The experiment was conducted in the spring of 2022. Participants were targeted via Facebook ads and directed to our Qualtrics survey. The experiment involved two stages, one in each language (the weak- and strong-FTR language). Each stage involved first taking a proficiency test and then reporting the perceived distance between the present and the future. The language proficiency tests in the experiment were used both to test for participants’ proficiency and to make the language salient. All the participants were asked questions in the two languages in which they declared being proficient (a within-subject design).

**Fig. 3.**
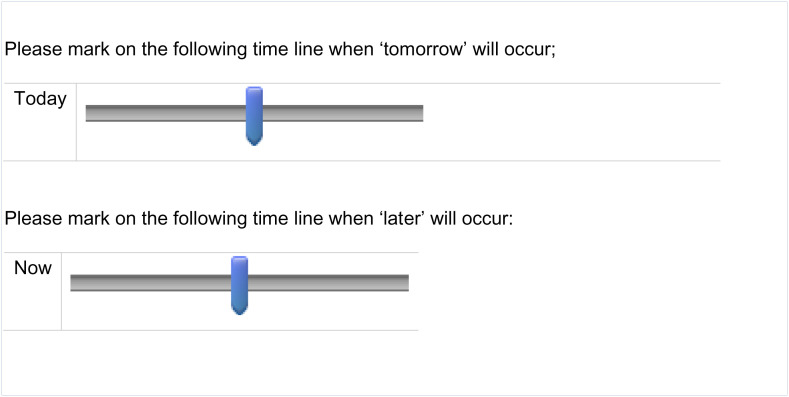
Sliders used in the experiment (study 3).

After being directed to our survey, participants were randomly assigned to whether questions were first asked in the weak- or strong-FTR language. Participants were first asked to take a language proficiency test in the randomly assigned language (either the weak- or strong-FTR language) and were then asked (in that same language), using a slider, to spatially mark the distance between the present and the future (participants were randomly assigned to mark the distance between now and later or between today and tomorrow). Fig. 3 demonstrates the sliders used in our experiment (in English).

As presented in the figure, the sliders we used did not include visible measurement units. Yet, we capture the perceived distance between the present and the future on a continuous variable (0 to 1) which represents the distance between the present and the future. After completing the first stage, participants were asked to take a second language proficiency test in the second language in which they declared being fluent (either the weak- or strong-FTR language based on the random assignment). They were then asked, again, using a slider, to spatially mark the distance between the present and the future. Participants who were randomly assigned in the first stage to mark the distance between “now” and “later” were asked in the second stage to mark the distance between “today” and “tomorrow” and vice versa.^||^ The words used in each language and proficiency tests are provided in *SI Appendix*.

**Fig. 4.**
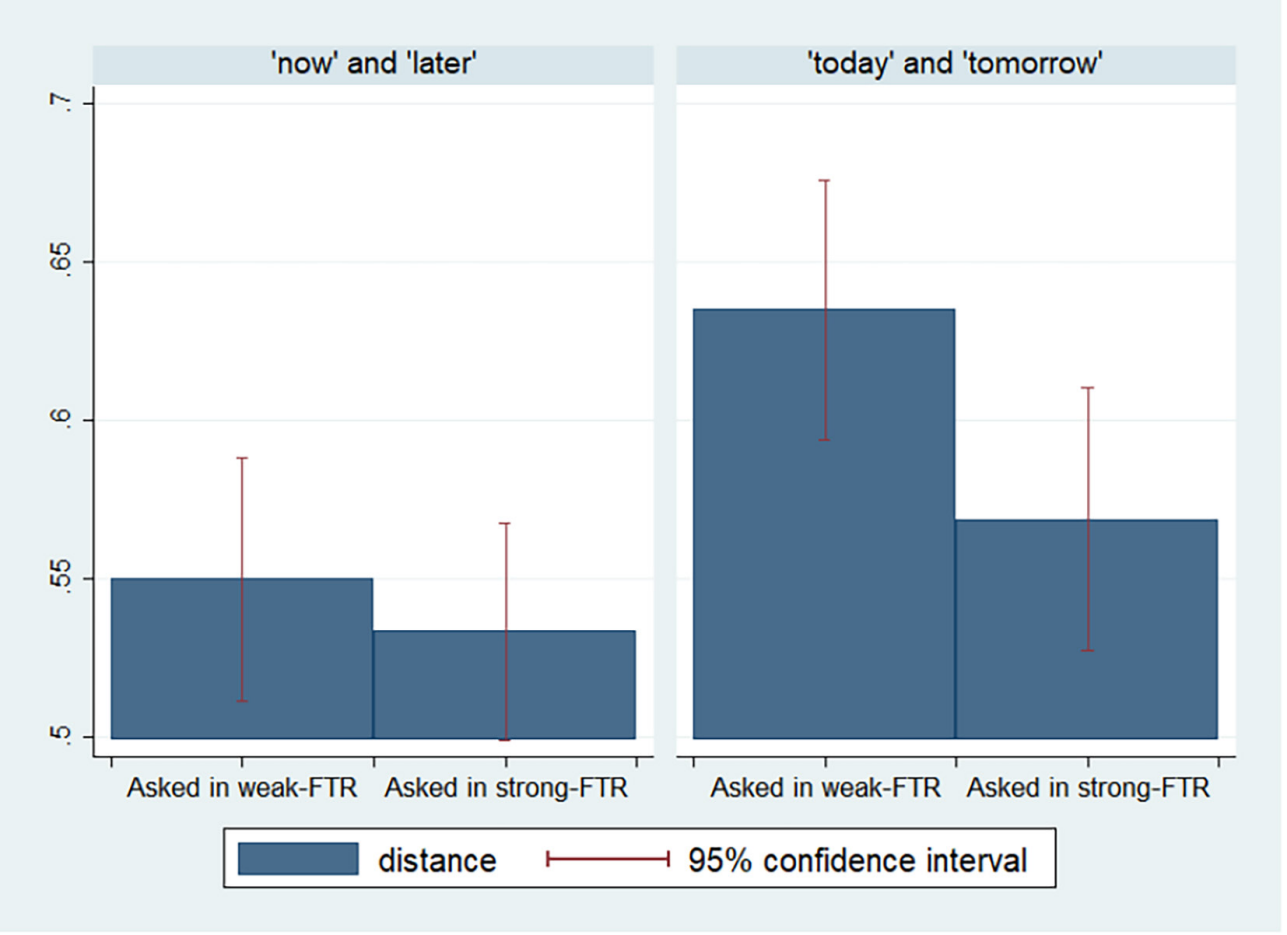
Perceived distance by experimental condition (study 3).

The final sample for the study included 570 participants who received scores higher than 6 of 9 in the two proficiency tests they took (participants with lower proficiency scores were not able to complete the study, and their responses were not recorded). In *SI Appendix*, Table S9, we report the sample characteristics. In *SI Appendix*, Table S10, we report the number of participants in each language pair we used in the experiment. Because each participant was asked two distance questions (one in each of the two languages), our final dataset includes 1,140 observations (two per participant). On average, the perceived distance between “today” and “tomorrow” was 0.60 (SD = 0.35, N = 570) and between now and later was 0.54 (SD = 0.31, N = 570). Fig. 4 presents the differences in the perceived distance by experimental condition.

### The Distance Hypothesis.

On average, the perceived distance between the present and the future is 0.59 (SD = 0.34, N = 570) when participants were addressed in the weak-FTR language and only 0.55 (SD = 0.33, N = 570) when participants were addressed in the strong-FTR language (*P* < 0.05). In other words, addressing people in strong-FTR languages lead them to mark the future as “less” distant.^**^

In Fig. 3, we present the perceived distance between the present and the future by experimental condition. In *SI Appendix*, Table S11, we report the results of OLS regression models predicting the perceived distance by whether questions were asked in the strong- or weak-FTR language and additional controls.

In all models, being asked the distance question in a strong-FTR language decreases the perceived distance between the present and the future by at least 0.03 (*P* < 0.05). Finally, even when person fixed-effects regression models are estimated (model 5), effects remain similar in magnitude and statistical significance. In other words, we observe a negative effect of being addressed in the strong-FTR language on the perceived distance between the present and the future, even within person. Note that the effects are driven by differences in the perceived distance between today and tomorrow and not by differences in the perceived distance between “now” and “later” which is a more “fuzzy” object.^††^

Taken together, the results of the experiment provide evidence that reject the distance hypothesis. Related findings were recently obtained by Robertson (26) who has found that English (strong-FTR language) speakers rated future outcomes as more proximal than Dutch speakers (weak-FTR language). One possible explanation is that the abstraction generated by using a weak- compared with a strong-FTR language leads people to view future events as more distant (40).

### The Precision Hypothesis.

**Fig. 5.**
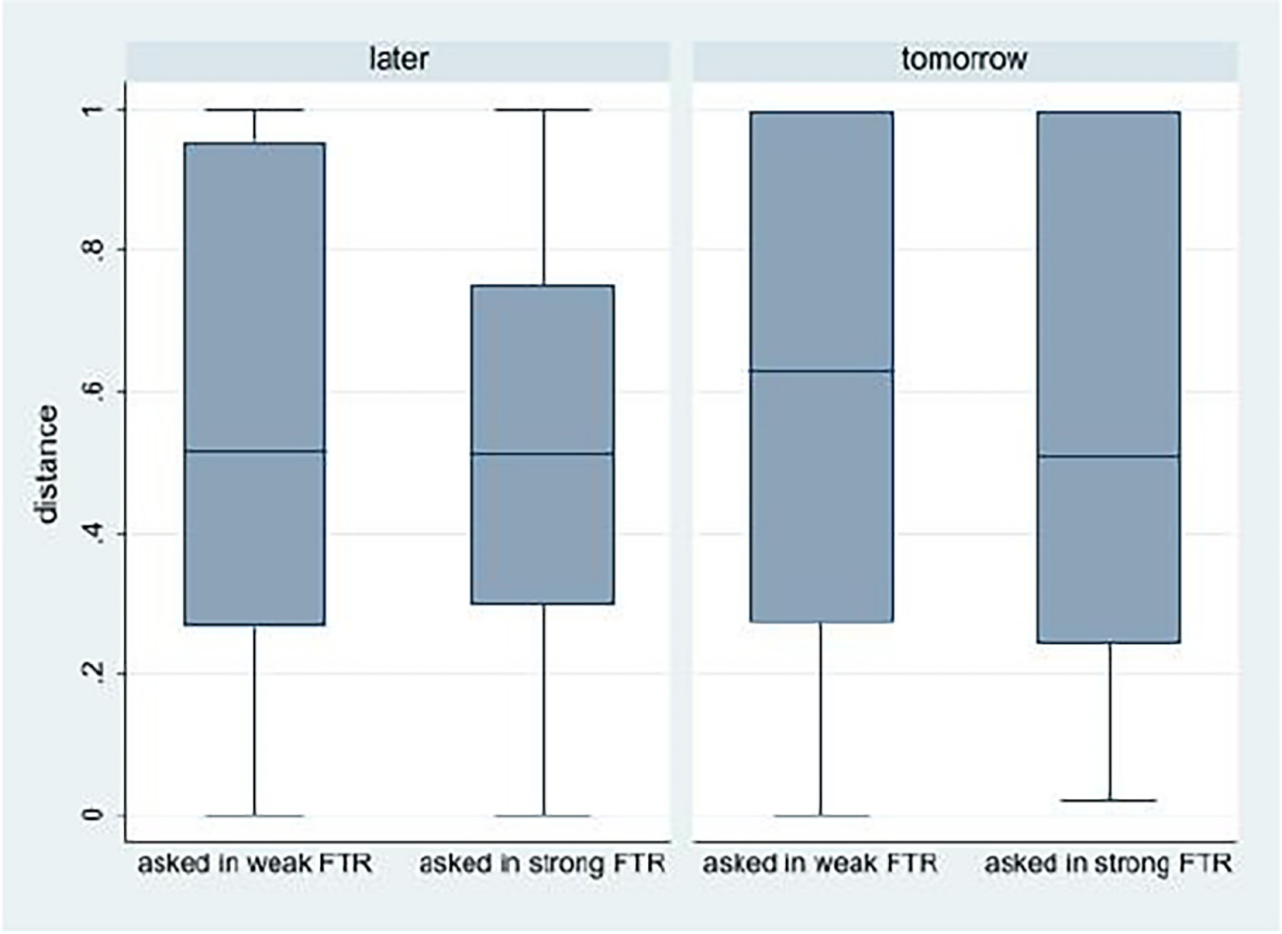
Perceived distance by experimental condition (study 3, box plots, all participants). The first and third quartiles of the perceived distance are represented by the upper and lower bounds of the boxes and the median by the line within the box. The range of the perceived distance is indicated by the “whiskers.”

We evaluate the precision of participants’ temporal beliefs (between participants) by estimating the variance in the perceived distances between the present and the future. Recall that the SD in the perceived distance between the present and the future is 0.34 when participants were addressed in a weak-FTR language and only 0.33 when participants were addressed in a strong-FTR language. In Fig. 5, we present the distributions of participants’ perceived distances by experimental condition.

The results of a Levene’s robust test statistic for the equality of variances show that when asked in a strong-FTR language the variance in participants’ perceived distance is smaller than when asked in a weak-FTR language (*P* < 0.01 for W0, W50, and W10).^‡‡^ Consistent with the precision hypothesis, the analysis further suggests that differences are in the perceived distance between now and later which is a more fuzzy object than the distance between today and tomorrow.

In order to better understand the effects on the variance, we calculated the median of the perceived distance between the present and the future by task (today and tomorrow or now and later) and the language in which participants were addressed. We then calculated for each response, the absolute value of difference between one’s own perceived distance between the present and the future and the median perceived distance.

Consistent with the precision hypothesis, on average, the difference between participants’ perceived distance and the median perceived distance between the present and the future is 0.30 (SD = 0.18, N = 570) when participants were addressed in the weak-FTR language and only 0.28 (SD = 0.18, N = 570) when participants were addressed in the strong-FTR language (*P* < 0.05). Results are robust to using the mean instead of the median perceived distance between the present and the future.

Finally, in *SI Appendix*, Table S12, we report the results of OLS regression models predicting the absolute value of difference between participants’ perceived distance and the median perceived distance between the present and the future by whether questions were asked in the strong- or weak-FTR language and additional controls. In all models, being asked the distance question in a strong-FTR language decreases the difference between participant’s perceived distance and the median perceived distance by at least 0.02 (*P* < 0.05). In fact, even when effects are estimated within participant (person fixed-effects), being addressed in a strong-FTR language results in a smaller difference compared with being addressed in a weak-FTR language (model 3, *P* < 0.1). Taken together, the findings of the experiment show that people express more precise temporal beliefs when addressed in a strong- compared with a weak-FTR language.

## Discussion

The results of our first experiment suggest that people addressed in languages in which the present and the future are marked more distinctly tend to value future events less than people addressed in languages in which the present and the future are similarly marked. In addition, people more fluent in languages in which the present and the future are marked more distinctly (compared with their fluency in languages in which the present and the future are similarly marked) tend to value future events less compared with people who are more fluent in languages in which the present and the future are similarly marked—regardless of the language in which they are being addressed. Finally, the effects of being addressed in languages in which the present and the future are marked more distinctly are stronger when participants are relatively more proficient in these languages compared with their proficiency in languages in which the present and the future are similarly marked. The results of our second experiment show similar effects on the tendency to delay gratification: When addressed in a strong-FTR language, people tend to prefer immediate gratification more than when addressed in a weak-FTR language. Finally, the results of our third experiment shed light on the mechanism and support the “precision hypothesis”: When people are addressed in languages in which the present and the future are marked more distinctly, they express more precise temporal beliefs compared with when they are addressed in languages in which the present and the future are similarly marked.

Our experimental designs enabled us to identify causality between the encoding of time in the language in which people are addressed and their tendency to discount future monetary payments, prefer immediate gratification, and express more precise temporal beliefs. Taken together, the results suggest that the grammatical structure of the language in which one is addressed activates different perceptions of time, resulting in different time preferences and behaviors. In order to provide stronger evidence for causality, our third experiment is a within-participant experiment.

Our studies have some limitations. FTR and modality (the strategies used to mark possible events) may correlate across languages. However, the EUROTYP project, which the FTR typology that we relied upon is based on, did not include any treatment of modality. We are not familiar with any quantitative typology of modality. Therefore, we cannot completely disentangle the effects of FTR from the effects of modality. Note also that the strong–weak-FTR distinction used in this study is not confounded by any of the linguistic features that appear in the World Atlas of Language Structures. In this atlas, there are 56 linguistic features that have complete data for all the languages used in this study. None of these features could be used to divide the languages used here into the two groups we use other than that of their FTR.^§§^

Our results suggest that the time-related schemas embedded in languages are easily and immediately activated; asking the same payment questions in a different language resulted in different time preferences for otherwise similar participants. Asking people to mark the distance between the present and the future in a different language resulted in differences in the precision of their temporal beliefs.

Languages both reflect and enforce time-related attitudes, preferences, and behaviors. The preferences of participants in the experiment were affected both by the encoding of time in the language in which they were addressed and by the encoding of time in the language in which they are more proficient. Thus, languages routinely and actively participate in enacting and maintaining schemas about time; whenever a language is spoken, the time preferences embedded in it are further reinforced, and behaviors follow accordingly.

## Supplementary Material

Appendix 01 (PDF)

## Data Availability

All data and codes have been deposited in OSF (https://osf.io/QCGPW/) (35).

## Footnotes

^*^It should be noted that almost all strong-FTR languages have some uses in which marking the future is not necessary, but these are mostly noncanonical or minor uses (19). Thus, for example, in English (a strong-FTR), one can say “I’m leaving tomorrow” or “the term begins next week.”

^†^These results can be found in *SI Appendix*.

^‡^We also tried to recruit participants who were fluent in Hindi (and in an additional weak-FTR language) but were not able to recruit enough bilingual research participants who were fluent in Hindi and in Mandarin, Dutch, or German.

^§^We suspect that the nature of the last task in the first stage affected the choices participants made in the second stage. Nonetheless, because the order in the first stage was affected by the first language in which participants were addressed, we cannot disentangle the effects of the order in the first stage and the effects of the language that was used in the second stage (that was by design, the other language in which the participant is fluent). For the preregistration of the within-person hypotheses, see https://osf.io/wzyfc.

^¶^When results are analyzed separately by the language pairs in which participants were addressed, we find that effects are similar in magnitude and are statistically significant for the following pairs: Mandarin and English (N = 91); similar but marginally significant for the following pairs: German and English (N = 103) and German and French (N = 94); similar but nonsignificant for the following pairs: Dutch and Spanish (N = 65) and German and Spanish (N = 79); and different in the direction and statistically nonsignificant in the following pairs: Dutch and English (N = 97) and Dutch and French (N = 78).

^#^We also tried to recruit participants who were fluent in Hindi (and in an additional weak-FTR language) but were not able to recruit enough bilingual research participants who were fluent in Hindi and in Mandarin, Dutch, or German.

^||^Hypotheses and design were registered at https://osf.io/pjqb5. Note that our original registered hypotheses and predictions focused only on the distance hypothesis. After they were rejected, we tested the precision hypothesis in our data and found support for it (https://osf.io/wzyfc).

^**^When results are analyzed separately by the language pairs in which participants were addressed, we find that effects are similar in magnitude and are marginally statistically significant for the following pairs: Dutch and Spanish (N = 154), German and Spanish (N = 104), and Mandarin and English (N = 108) and similar but nonsignificant for the following pairs: Dutch and English (N = 192), Dutch and French (N = 188), German and English (N = 206), German and French (N = 140), and Mandarin and French (N = 48).

^††^We additionally tested whether results were predicted by the actual time of the day for the participant by including the time stamps in the main regression models (adjusted to participant’s location). The results are robust to the inclusion of time stamps. When included in the main regression model, the effect of the time (h) of the day is statistically nonsignificant (both when included as a continuous variable or as a set of binary variables). The main experimental condition remains similar in magnitude and statistical significance (*P* < 0.05).

^‡‡^When results are analyzed separately by the language pairs in which participants were addressed, we find that in all language pairs, the variance is smaller when participants were addressed in the strong compared with the weak-FTR language. The variance is significantly smaller in the strong-FTR compared with the weak-FTR language in the following pairs: German and Spanish (N = 104); is smaller but the difference is only marginally statistically significant in the following pairs: German and English (N = 206) and Dutch and Spanish (N = 154); and is smaller but the difference is not statistically significant in the following pairs: Dutch and English (N = 192), Mandarin and English (N = 108), Dutch and French (N = 188), German and French (N = 140), and Mandarin and French (N = 48).

^§§^We are grateful to the anonymous reviewer who brought this evidence to our attention.
