# Supplementary material for: Languages and future-oriented economic behavior—Experimental evidence for causal effects
Source: Proc Natl Acad Sci U S A. 2023 Feb 6;120(7):e2208871120. doi: 10.1073/pnas.2208871120 (PMC9964034; doi:10.1073/pnas.2208871120)
Supplement: Supplementary file 1 — Appendix 01 (PDF) [file pnas.2208871120.sapp.pdf]

SI Appendix: Tables

Table S1: OLS Regression Models Predicting Lowest Accepted Delayed Payment

	(1)	(2)	(3)	(4)	(5)
Asked in Strong FTR	0.303***	0.298**	0.179*	0.295**	0.293**
	(0.091)	(0.091)	(0.094)	(0.095)	(0.092)
Proficiency in the Addressing Language	-0.252***	-0.248***	-0.343***	-0.232***	-0.219***
	(0.039)	(0.040)	(0.045)	(0.041)	(0.040)
Strong Weak Proficiency Gap		0.056	-0.122*	0.054	0.047
		(0.041)	(0.050)	(0.049)	(0.048)
Asked in Strong FTR X Strong Weak Proficiency Gap			0.347***		
			(0.088)		
Female				0.159ǂ	0.177*
				(0.090)	(0.090)
African American				0.514ǂ	0.571ǂ
				(0.312)	(0.309)
Hispanic				-0.148	-0.154
				(0.194)	(0.188)
Asian				-0.288*	-0.334**
				(0.118)	(0.115)
Other				0.019	0.007
				(0.302)	(0.301)
College Graduate				-0.175	-0.200
				(0.132)	(0.132)
Language Pairs Dummies				Y	
Strong-FTR Genus Indic					0.109
					(0.153)
Strong-FTR Genus Romance					0.044
					(0.103)
Weak-FTR Genus Indic					-0.092
					(0.110)
Constant	5.731***	5.671***	6.478***	5.685***	5.697***
	(0.307)	(0.318)	(0.363)	(0.366)	(0.373)
N	523	523	523	509	509
Adjusted R-square	0.071	0.073	0.100	0.113	0.100

Robust standard errors in parentheses; ǂ p<0.1 * p<0.05 ** p<0.01 ***p<0.001

Table S2: Ordered Logit Regression Models Predicting Lowest Accepted Delayed Payment

	(1)	(2)	(3)	(4)	(5)
Asked in Strong FTR	0.574***	0.639***	0.391**	0.705***	0.673***
	(0.159)	(0.156)	(0.167)	(0.165)	(0.158)
Proficiency in the Addressing Language	-0.588***	-0.596***	-0.814***	-0.645***	-0.616***
	(0.074)	(0.070)	(0.087)	(0.077)	(0.078)
Strong Weak Proficiency Gap		0.081	-0.274***	0.011	0.001
		(0.062)	(0.083)	(0.075)	(0.073)
Asked in Strong FTR X Strong Weak Proficiency Gap			0.719***		
			(0.144)		
Female				0.002	0.019
				(0.163)	(0.161)
African American				0.642	0.713 †
				(0.414)	(0.392)
Hispanic				0.328	0.369
				(0.359)	(0.366)
Asian				-0.631**	-0.661**
				(0.236)	(0.223)
Other				1.002 †	0.901 †
				(0.516)	(0.504)
College Graduate				-0.322	-0.325
				(0.211)	(0.210)
Language Pairs Dummies				Y	
Strong-FTR Genus Indic					-0.289
					(0.298)
Strong-FTR Genus Romance					-0.232
					(0.181)
Weak-FTR Genus Indic					-0.146
					(0.192)
cut1	-5.070***	-5.158***	-6.999***	-6.183***	-6.124***
	(0.583)	(0.550)	(0.701)	(0.709)	(0.734)
cut2	-4.511***	-4.620***	-6.441***	-5.608***	-5.556***
	(0.574)	(0.542)	(0.689)	(0.700)	(0.724)
cut3	-4.154***	-4.286***	-6.095***	-5.245***	-5.199***
	(0.567)	(0.536)	(0.682)	(0.693)	(0.717)
cut4	-3.919***	-4.070***	-5.872***	-5.014***	-4.972***
	(0.561)	(0.531)	(0.677)	(0.688)	(0.711)
cut5	-3.223***	-3.459***	-5.243***	-4.362***	-4.333***
	(0.545)	(0.518)	(0.661)	(0.677)	(0.698)
cut6	-2.757***	-3.082***	-4.858***	-3.970***	-3.950***
	(0.533)	(0.510)	(0.653)	(0.669)	(0.689)
cut7	-1.908***	-2.494***	-4.261***	-3.340***	-3.330***
	(0.529)	(0.509)	(0.651)	(0.666)	(0.681)
cut8	-1.664**	-2.355***	-4.120***	-3.207***	-3.198***
	(0.528)	(0.508)	(0.650)	(0.666)	(0.681)
cut9	-1.088*	-2.084***	-3.846***	-2.923***	-2.917***
	(0.535)	(0.510)	(0.650)	(0.668)	(0.681)
cut10		-1.575***	-3.330***	-2.390***	-2.388***
		(0.525)	(0.658)	(0.677)	(0.691)
N	523	565	565	551	551
Pseudo R-square	0.032	0.033	0.043	0.055	0.049

Robust standard errors in parentheses; † p<0.1 * p<0.05 ** p<0.01 ***p<0.001

Table S3: Balancing Tests (experiement 1)

	Asked in Weak FTR	Asked in Strong FTR	t-test p-value
	(1)	(2)	(1)-(2)
Strong FTR Language: English	0.484	0.507	0.588
	(0.029)	(0.030)	
Strong FTR Language: French	0.131	0.185	0.083*
	(0.020)	(0.023)	
Strong FTR Language: Hindi	0.183	0.123	0.047**
	(0.023)	(0.020)	
Strong FTR Language: Spanish	0.201	0.185	0.632
	(0.024)	(0.023)	
Weak FTR Language: Dutch	0.270	0.275	0.884
	(0.026)	(0.027)	
Weak FTR Language: German	0.453	0.446	0.856
	(0.029)	(0.030)	
Weak FTR Language: Mandarin	0.277	0.279	0.954
	(0.026)	(0.027)	
Strong FTR proficiency score	8.076	8.152	0.408
	(0.064)	(0.065)	
Weak FTR proficiency score	7.453	7.511	0.545
	(0.067)	(0.068)	
N	289	276	

Robust standard errors in parentheses. * p<0.1 ** p<0.05 ***p<0.01

Table S4: Tobit Regression Models Predicting Lowest Accepted Delayed Payment

	Excluding French	Excluding Hindi
	(1)	(2)
Asked in Strong FTR	0.544***	0.405**
	(0.161)	(0.150)
Proficiency in the Addressing Language	-0.390***	-0.409***
	(0.069)	(0.067)
Strong Weak Proficiency Gap	0.120*	0.080
	(0.061)	(0.057)
Constant	6.291***	6.599***
	(0.525)	(0.508)
Sigma	2.139***	1.948***
	(0.200)	(0.177)
N	443	443
Pseudo R-sq	0.031	0.031

Robust standard errors in parentheses; ⁱ p<0.1 * p<0.05 ** p<0.01 ***p<0.001

Table S5: TOBIT Regression Models Predicting Lowest Accepted Delayed Payment, Excluding English

	(1)	(2)	(3)	(4)	(5)
Asked in Strong FTR	0.441*	0.476*	0.333	0.455*	0.462*
	(0.215)	(0.215)	(0.211)	(0.209)	(0.21)
Proficiency in the Addressing Language	-0.611***	-0.613***	-0.813***	-0.558***	-0.521***
	(0.094)	(0.093)	(0.107)	(0.093)	(0.093)
Strong Weak Proficiency Gap		0.167¡	-0.228¡	0.153	0.152
		(0.094)	(0.133)	(0.093)	(0.094)
Asked in Strong FTR X Strong Weak Proficiency Gap			0.856***		
			(0.213)		
Female				0.338	0.389¡
				(0.213)	(0.217)
African American				1.051	1.108
				(0.775)	(0.79)
Hispanic				-0.634	-0.541
				(0.402)	(0.4)
Asian				-0.879***	-0.847**
				(0.261)	(0.26)
Other				0.395	0.328
				(0.487)	(0.495)
College Graduate				-0.586*	-0.581¡
				(0.293)	(0.297)
Language Pairs Dummies				Y	
Strong-FTR Genus Indic					0.026
					(0.253)
Weak-FTR Genus Indic					-0.557*
					(0.24)
Constant	7.939***	7.895***	9.510***	8.642***	8.293***
	(0.692)	(0.688)	(0.8)	(0.786)	(0.754)
/					
Sigma	2.481***	2.449***	2.299***	2.058***	2.173***
	(0.315)	(0.311)	(0.29)	(0.263)	(0.278)
N	260	260	260	254	254
Pseudo R-square	0.058	0.062	0.082	0.116	0.101

¡ p<0.1 * p<0.05 ** p<0.01 ***p<0.001

Table S6: Descriptive Statistics (Study 2)				
	mean	sd	min	max
Female	0.577		0	1
Non binary	0.010		0	1
High school or less	0.119		0	1
Age	48.725	15.064	16	91
Proficiency: strong FTR	8.722	0.573	5	9
Proficiency: weak FTR	8.455	0.837	5	9
Proficiency gap (strong -weak FTR)	0.268	0.879	-3	4
N=598				

Table S7: Number of Participants by Language Pairs and Experimental Condition (Study 2, first choice)

	asked in weak-FTR		asked in strong-FTR	
	Obs	Proportion	Obs	Proportion
Language Pairs				
English-Dutch	46	0.20	51	0.15
English-German	44	0.19	59	0.17
English-Mandarin	42	0.18	49	0.14
French-Dutch	41	0.18	37	0.11
French-German	39	0.17	55	0.16
Spanish-Dutch	22	0.09	34	0.10
Spanish-German	23	0.10	56	0.16
	234		341	

Table S8: OLS Regression Models Predicting Choosing Enjoyable Task First				
	(1)	(2)	(3)	(4)
Asked in Strong FTR	0.071 ɨ	0.070 ɨ	0.068 ɨ	0.066 ɨ
	(0.040)	(0.039)	(0.040)	(0.040)
Set B		0.072 ɨ	0.072 ɨ	0.072 ɨ
		(0.039)	(0.039)	(0.039)
Strong Weak Proficiency Gap				-0.038
				(0.024)
Language Dummies			Y	Y
Constant	0.603***	0.567***	0.552***	0.559***
	(0.030)	(0.036)	(0.050)	(0.050)
N	598	598	598	598
Adjusted R-sq	0.004	0.008	0.004	0.006

Standard errors in parentheses

ɨ p<0.1 * p<0.05 ** p<0.01 ***p<0.001

Table S9: Descriptive Statistics (Study 3)				
	mean	sd	min	max
Female	0.809	0.393		
Non binary	0.005	0.724		
High school or less	0.082	0.275		
Age	47.174	13.696	20	122
Proficiency: strong FTR	8.830	0.432	6	9
Proficiency: weak FTR	8.581	0.666	6	9
Proficiency gap (strong -weak FTR)	0.249	0.782	-3	3

N=570

Table S10: Number of Participants by Language Pairs (Study 3)

	N
Language Pairs	
English-Dutch	96
English-German	103
English-Mandarin	54
French-Dutch	94
French-German	70
French-Mandarin	24
Spanish-Dutch	77
Spanish-German	52
	570

Table S11: OLS Regression Models Predicting the Perceived Distance between the Present and the Future

	(1)	(2)	(3)	(4)	(5)	(6)
Asked in strong FTR	-0.041***	-0.032*	-0.033*	-0.033*	-0.035*	-0.041*
	(0.012)	(0.014)	(0.014)	(0.014)	(0.014)	(0.019)
Task ('tomorrow')	0.060***	0.060***	0.060***	0.060***	0.060***	0.060***
	(0.012)	(0.012)	(0.013)	(0.013)	(0.013)	(0.018)
Proficiency in asked		-0.037*	-0.036*	-0.036 ᵻ	-0.030	0.000
		(0.018)	(0.018)	(0.019)	(0.019)	(0.025)
Proficiency gap :(strong-weak FTI		0.006	0.006	0.003	0.004	
		(0.016)	(0.016)	(0.017)	(0.018)	
First choice			0.011	0.011	0.011	
			(0.013)	(0.013)	(0.013)	
Age					0.002 ᵻ	
					(0.001)	
Female					0.029	
					(0.033)	
Non-binary					-0.065	
					(0.127)	
Non-white					-0.022	
					(0.043)	
High school or less					0.061	
					(0.051)	
Constant	0.563***	0.874***	0.867***	0.869***	0.708***	0.559*
	(0.015)	(0.160)	(0.161)	(0.165)	(0.174)	(0.217)
Language dummies				Y	Y	
Person Fixed effects						Y
N	1140	1140	1140	1140	1140	1140

Standard errors in parentheses, clustered by person

ᵻ p<0.1 * p<0.05 ** p<0.01 ***p<0.001

Table S12: OLS Regression Models Predicting the Difference between Participants' Perceived Distance and the median Perceived Distance between Present and Future

	(1)	(2)	(3)
Asked in strong FTR	-0.019*	-0.032*	-0.023 ǂ
	(0.009)	(0.015)	(0.013)
Task ('tomorrow')	0.057***	0.043**	0.056***
	(0.008)	(0.015)	(0.012)
Proficiency in Asked	0.001	0.001	0.019
	(0.010)	(0.010)	(0.013)
Proficiency gap :(strong-weak FTR)	0.004	0.004	
	(0.008)	(0.008)	
First choice	-0.010	-0.010	-0.010
	(0.008)	(0.008)	(0.012)
Asked in strong X 'tomorrow'		0.026	
		(0.024)	
Person fixed effects			Y
Constant	0.261**	0.265**	0.109
	(0.085)	(0.085)	(0.115)
N	1140	1140	1140

Standard errors in parentheses, clustered by person

ǂ $p<0.1$ * $p<0.05$ ** $p<0.01$ *** $p<0.001$

SI Appendix: Experimental Materials

Fluency tests

Fluency tests for all seven languages as well as the payment options for study 1 can be found in our Web Appendix at https://ianayres.yale.edu/sites/default/files/files/Survey%20Question%20Appendix.pdf

Cross-Linguistic Semantics

Words Used in Study 1			
English	we pay $3 today		we will pay $3.05 in a week
French	Nous vous payons $3 aujourd'hui		Nous vous paierons $3.05 dans 1 semaine
Spanish	Pagamos $3 actualmente		Pagaremos $3.05 dentro de 1 semana
Hindi	हम आज $3 का भुगतान		करेंगे हम 1 सप्ताह में $3.05 का भुगतान करेंगे।
German	wir zahlen entweder heute 3 $		wir zahlen in einer Woche 3,05 $
Dutch	we betalen $3 vandaag		we betalen $3.05 over 1 week
Mandarin	我们在当日支付$3		者我们将在1周内支付$3.05

Words Used in Study 2		
English	you will be asked	Which task will you start with?
French	vous devrez	Quelle tâche allez-vous faire en premier?
Spanish	se te pedirá	¿Con qué tarea empezarás?
Hindi	आपसे कहा जाएगा	आप किस कार्य से शुरुआत करेंगे?
German	werden Sie aufgefordert	Mit welcher Aufgabe werden Sie beginnen?
Dutch	wordt je gevraagd	Met welke taak wil je beginnen?
Mandarin	我们将要求您	您将从哪个任务开始？

Words Used in Study 3				
English	today	tomorrow	now	later
French	aujourd’hui	demain	maintenant	plus tard
Spanish	Hoy	Mañana	Ahora	Más tarde
German	heute	morgen	jetzt	später
Dutch	vandaag	morgen	nu	later
Mandarin	今天	明天	现在	以后
